# Ethnic diversity and inclusiveness among medical residents in the Netherlands: results from a single-centered survey study

**DOI:** 10.1186/s12909-025-06878-5

**Published:** 2025-02-25

**Authors:** Linda Al-Hassany, Rianne J. Zaal, Karen M. Stegers-Jager, Adrienne A. M. Zandbergen

**Affiliations:** 1 Department of Internal Medicine, Erasmus MC University Medical Center, PO Box 2040, Rotterdam, CA 3000 The Netherlands; 2 Department of Hospital Pharmacy, Erasmus MC University Medical Center, Rotterdam, The Netherlands; 3 Radboudumc Health Academy, Radboud University Medical Center, Nijmegen, The Netherlands

**Keywords:** Medical residents, Diversity, Inclusiveness, Ethnicity, Specialty

## Abstract

**Purpose of study:**

Despite the recognized importance of an ethnically diverse healthcare workforce, the current population of medical specialists does not adequately reflect our society. To further unravel how and at which stages of the career path such diversity loss occurs, we studied ethnic diversity and perceptions of inclusiveness among medical residents.

**Materials and methods:**

We conducted a cross-sectional study among all residents of Erasmus Medical Center in the highly multicultural city of Rotterdam, the Netherlands. An online survey was distributed, in which we inquired about (i) ethnic diversity and (ii) perceptions of inclusivity. The latter outcome includes sense of belonging to the team of supervisors, measured by perceived level of resemblance between residents and their supervisor(s), while focusing on ethnic, cultural, and socioeconomic levels. Residents from different specialties were divided into four groups, according to their medical specialty, i.e., surgical specialties, internal medicine specialties, overall diagnostic/supportive specialties, and family medicine & intellectual disability medicine. Descriptive statistics were applied.

**Results:**

From the total of 986 invited residents, 493 (50.0%) participated (median age 32 years [IQR 30–34]), consisting of 346 (70.2%) females. Results showed that the majority, 335 (68.2%) were of Dutch origin, 90 (18.3%) were children of migrants, and 66 (13.4%) were migrants. We observed notable differences across medical specialties, with the highest degree of ethnic diversity in surgical specialties. Except for residents from supportive specialties, residents from other specialties who are (children of) migrants reported significantly more often that they experienced differences on ethnic and/or cultural levels with their supervisors than residents of Dutch origin.

**Conclusion:**

While (children of) migrants were underrepresented in this cohort, especially given the demographic distribution of the region of Rotterdam, a comparison of our results with previously published census data on medical students cohorts indicates no substantial loss of ethnic diversity in the transition from medical student to residency. Yet, these groups scored lower on questions related to sense of belonging.

**Clinical trial number:**

Not applicable.

**Supplementary Information:**

The online version contains supplementary material available at 10.1186/s12909-025-06878-5.

## Practice points

In the largest Dutch academic medical center, situated in one of the most multicultural areas in the Netherlands, the proportions of residents who are children of migrants and migrants are relatively high, yet do not form an adequate representation of the region.

The degree of diversity is the highest among surgical specialties, and overall, there is no indication for loss in diversity in the transition from medical student to resident.

Residents who are migrants or children of migrants score lower on questions regarding the perceived level of resemblance between residents and their supervisor(s).

No clear association was observed between degree of diversity and level of perceived resemblance.

To further improve sense of belonging to the team during residency training and fairness and equality in the transition of young medical doctors to medical specialists, further intersectional research remains warranted.

**Supplementary Information:**

The online version contains supplementary material available at 10.1186/s12909-025-06878-5.

## Introduction

Like most Western countries, migration is rising in the Netherlands, resulting in a more culturally diverse society [1, 2], with 25.2% of the population in the Netherlands being of non-Dutch origin in 2022 [2]. Consequently, there is an urgent need to diversify the healthcare workforce to serve and represent the patient population and prevent health-related societal inequities [3, 4]. Nowadays, patients from minority ethnicities still encounter more morbidity and mortality compared to non-minorities [5]. At the same time, diversity among physicians has been shown to improve access and reduce disparities among minority patients [6, 7]. More specifically, physician-patient (race) concordance does not only improve therapy adherence and compliance [8] but also mitigates provider implicit bias and improves the quality of care, patient satisfaction, communication and interactions between physicians and patients [4, 9–12]. In addition, diversity among healthcare professionals has been hypothesized to play an essential role in recruiting underrepresented individuals to clinical trials [13].

Unfortunately, the present situation is markedly different from the ideal scenario. Current physicians are not an adequate reflection of our society with regard to ethnic diversity, ascribed to the so-called “leaky pipeline” phenomenon. This describes the gradual loss of medical students from minority ethnicities (beginning already at an early stage in the admission procedures for medical school [14]), as well as early-career physicians from minority ethnicities during the course of further medical training and specialization [15]. This loss is typically accompanied by an underrepresentation of groups with a lower socioeconomic status [16]. More specifically, previous research demonstrated that Dutch medical students without a migration background receive higher grades than students with a migration background, especially during their clerkships [17]. Also, ethnic minority students experience higher perceptions of unfair treatment and lower trust in their supervisors during their clinical training [18]. While perceptions of inclusiveness and sense of belonging play a pivotal role in mental health and personal or academic successes [19], their absence contributes to discrepancies with respect to career prospects between young medical doctors with and without a migration background [18]. Accordingly, a Dutch cohort study published in 2023, based on Statistics Netherlands data, revealed a national gradual loss of medical students and doctors with an ethnic minority background during different phases of training [15]. This study showed that physicians with a migration background – especially women – have reduced chances to become a hospital specialist, thereby further increasing their underrepresentation.

Previous research primarily focused on medical students and specialists rather than career steps in between. The current degree of ethnic diversity among medical residents (specialists in training) is unclear. However, this information is essential in order to further unravel how and at which stages of the career path the “leaky pipeline” occurs.

Therefore, the current study explored the degree of diversity of residents in the largest Dutch academic medical center, located in one of the most multicultural areas in the Netherlands – namely, the city of Rotterdam and its surroundings, where 56.5% of the inhabitants are non-Dutch [20]. Considering the broad range of characteristics that diversity encompasses, the current study primarily focuses on ethnic diversity and the representation of ethnic minorities. We also studied inclusiveness among residents, including sense of belonging to the team of supervisors. The latter was measured by identifying the perceived level of resemblance between residents and their supervisor(s), while focusing on ethnic, cultural, and socioeconomic levels and accounting for differences between specialties. These results are important to create urgency concerning the degree of representation of certain ethnic groups among residents. Moreover, this information is essential to design further studies on which factors contribute to the lagging diversity among medical specialists, for example self-selection, current selection criteria and procedures for medical specialties, drop-out rates and level of inclusiveness.

## Materials and methods

The current study is a prospective cross-sectional online survey study among the residents of all 33 different medical specialties in the Erasmus Medical Center (Erasmus MC) in Rotterdam, The Netherlands. The study started in April 2023, and questionnaires were closed in October 2023. Initially, a single online questionnaire was sent by e-mail, and four reminders were sent.

### Study population

Eligible participants were residents working at the Erasmus MC in Rotterdam, The Netherlands, with a valid Erasmus MC e-mail address. No exclusion criteria were applied. A total of 986 residents were invited.

The residents were divided into four groups, according to their medical specialty: (i) *Surgical specialties* (Anesthesiology, Cardiothoracic Surgery, Emergency Medicine, Gynecology & Obstetrics, Oral and Maxillofacial Surgery, Neurosurgery, Ophthalmology, Orthopedics, Otorhinolaryngology (ENT - Ear, Nose, and Throat), Plastic Surgery, General Surgery, Urology); (ii) *Internal medicine specialties* (Cardiology, Clinical Geriatrics, Dermatology, Gastroenterology, General Internal Medicine, Neurology, Pediatrics, Pulmonary Diseases and Tuberculosis, Psychiatry, Rheumatology); (iii) An overall group of *Diagnostic*/*supportive specialties* (Clinical Chemistry, Clinical Genetics, Clinical Physics, Hospital Pharmacy, Medical Microbiology, Nuclear Medicine, Pathology, Radiology, Radiotherapy); and (iv) *Family medicine & intellectual disability medicine* (Intellectual Disability Medicine, General Practice (Family Medicine)).

### Study variables

A questionnaire was developed specifically for this study (Supplemental Materials file 2). The questionnaire has not undergone formal validation or piloting; however, it was extensively tested and reviewed by the research team prior to distribution. This online questionnaire included queries on baseline characteristics: medical specialty of training, age and sex. Origin was an objective measure of ethnic diversity and based on the residents own and their parents’ country of birth. Second, perceptions of inclusivity were explored by examining sense of belonging as a subjective outcome, starting with an assessment of the perceived level of resemblance between residents and their supervisor(s). Therefore, we first asked to what extent respondents identify with their supervisor(s) at work (they were asked to provide a discrete score between 1 and 10, with 10 indicating complete identification). If any score below 10 was given, we subsequently further explored their sense of belonging and asked: “In which level(s) do you experience differences?“. Participants were allowed to choose one or more of the following areas that were not further explained: ethnicity, culture, or socio-economic status or other (with required specification). In addition, to further explore inclusivity, a question about which ethnicity or ethnicities the resident identifies with, allowing for multiple entries, was included; participants were asked to rank these in order of level of identification from most to least. Lastly, we asked whether the residents have higher-educated parent(s)/guardian(s) and a medical doctor as parent(s)/guardian(s).

Ethnicity was based on the new classification of origin according to the Statistics Netherlands, which consists of two parts: (i) born in the Netherlands and (ii) country of origin (with a distinction made between inside or outside the Netherlands and a subclassification of the latter group into inside Europe or outside Europe) [21]. Thus, the initial step determines if a participant was born in the Netherlands or outside the Netherlands. The subsequent step involves exploring the birth country of the parents, i.e., the Netherlands or outside the Netherlands. Migrants are born outside the Netherlands, and children of migrants themselves are born in the Netherlands and have at least one parent born outside the Netherlands.

### Medical ethical procedures

The study has been approved by the Medical Research Ethics Committee of Erasmus Medical Center, Rotterdam, the Netherlands (MEC-2022-0826). Written informed consent was first obtained from all participants after a written explanation of the study. The participant was shown the questionnaire only after providing consent.

Personal data were handled confidentially in compliance with the EU General Data Protection Regulation (GDPR) and the Dutch Act on Implementation of the General Data Protection Regulation (UAVG). All questionnaires were sent to the participants by using the survey software LimeSurvey. Answers were automatically stored in GemsTracker (GEneric Medical Survey Tracker), which is a software developed at Erasmus Medical Center allowing secure collection of data.

### Statistical analyses

We described the distribution of ethnicity of residents in the four groups of different specialties, along with their age and sex. To achieve a comprehensive and representative overview of the ethnic backgrounds among all residents in our cohort, we aimed for the highest possible response rate rather than performing a sample size calculation.

Descriptive statistics, i.e., Chi-squared tests, were applied to study whether the origin of respondents differed across the four groups of specialties. Further, we tested for differences in the level of perceived resemblance between supervisors and residents, while stratifying by origin and specialty. Sense of belonging was further explored by stratifying by levels on which discrepancies were experienced (i.e. ethnic, cultural, and/or socio-economic). Additionally, we tested whether having higher-educated parent(s)/guardian(s) and having a medical doctor as parent(s)/guardian(s) differed across ethnicities. Cramér’s V was calculated to provide effect sizes of the Chi-squared test results, with a value of ≥ 0.6 indicating a strong association. Adjusted standardized residuals (ASRs) were reported to identify which subgroups drove the significant results. If these residuals were higher than 1.96 or lower than − 1.96, we considered the observed count to be significantly larger than or less, respectively, than would be expected.

Two-tailed P-values of ≤ 0.05 were considered to be statistically significant. No corrections for multiple testing have been made, considering the explorative nature of the study. All data were handled and analyzed blinded for the respondent’s identity using SPSS 29.0.1.0 for Windows (SPSS Inc., Chicago, IL, USA). Graphs were created using GraphPad Prism version 8.0.1 for Windows (GraphPad Software, Boston, Massachusetts USA).

## Results

The response rate was 50.0%, with 493 completed questionnaires of 986 invitations. For 23 (2.3%) residents, the e-mail (either the initial invitation or reminder) bounced back as undeliverable. An overview of the distribution of the respondents among the different specialties, along with their age and sex, is provided in Table 1. An overview of the classification by origin of all respondents, stratified by specialty, is provided in Table 2. Overall, the majority (68.2%) of residents was of Dutch origin (born in the Netherlands and both parents born in the Netherlands), followed by residents who were children of migrants (18.3%, born in the Netherlands and either one or both parents born outside the Netherlands), and migrants (13.4%, born outside the Netherlands).

The proportion of the origins of respondents differed significantly across different specialties (χ^2^ (df = 6, *N* = 491) = 12.7, *P* = 0.047, *V* = 0.11 (small effect)). This was mainly driven by the lower proportion of residents with Dutch origin among the surgical specialties (59.2% vs. 68.2% in the total study population, ASR − 2.5) and a higher proportion of residents with Dutch origin among the diagnostic/supportive specialties (80.9%, ASR 2.0) than expected. In addition, the surgical specialties had a higher proportion of migrants than expected (20.8% vs. 13.4% in the total study population, ASR 2.7). However, the distribution of origin classifications differed slightly across different specialties. Overall, the proportion of children of migrants was higher than that of migrants, except in surgical and diagnostic/supportive specialties. Further, diagnostic/supportive specialisms had the highest proportion of residents of Dutch origin (80.9%), followed by internal medicine specialties (70.4%), family & intellectual disability medicine (69.1%), and finally, surgical specialties (59.2%) (Table 2).

In Table 3, the number of residents of whom at least one parent/guardian completed higher education and the number of whom at least one parent/guardian is/was working as a medical doctor are presented. The proportion of having a higher-educated parent(s)/guardian(s) was significantly different across the ethnicities (χ^2^ (df = 2, *N* = 489) = 8.6, *P* = 0.014, *V* = 0.13 (small effect)), mainly driven by the lower proportion of children of migrants with higher-educated parent(s)/guardian(s) (61.8%, ASR − 2.9) than expected. Migrants reported most often to have parent(s)/guardian(s) working as a medical doctor. The proportion of having a medical doctor as parent(s)/guardian(s), however, did not significantly differ across the ethnicities (χ^2^ (df = 2, *N* = 490) = 1.7, *P* = 0.417, *V* = 0.06).

### Results related to “Perceptions of inclusivity”

In Table 4a, the median scores on how well residents can identify with their supervisor are presented, showing the lowest median scores among migrants, across all specialties.

In Table 4b, the proportion of residents who rank Dutch origin first from the list of ethnicities they identify with, are presented. These results indicate that almost all residents with Dutch origin identify most with the Dutch ethnicity, followed by children of migrants and migrants. Among residents of diagnostic/supportive specialties and family medicine & intellectual disability medicine, fewest migrants rank Dutch origin as the ethnicity that they identify with the most.

**Table 1:** An overview of the number of respondents with their median age and proportion of females, stratified by specialty

	Respondents (*n*, % of total respondents)		Age in years^1^ (median [IQR])		Female^1^ (*n*, % of respondents within the concerning specialty)	
Surgical	120	(24.3%)	32	[30–34]	76	(63.3%)
Internal medicine	159	(32.3%)	33	[31–35]	104	(65.4%)
Diagnostic/supportive	47	(9.5%)	33	[31–36]	36	(76.6%)
Family & intellectual disability medicine	167	(33.9%)	31	[29–33]	130	(77.8%)
TOTAL	493		32	[30–34]	346	(70.2%)

^1^ Data from two respondents in total are missing due to an incorrectly provided or absent answer to the questions

**Table 2:** An overview of the distribution of the classification by origin of respondents, stratified by specialty. The left percentages listed for each specialty indicate the proportion of individuals within that specialty relative to the total number of people who are of Dutch origin, migrant, or child of migrant(s). The right percentages listed indicate the proportion of individuals with a certain classification by origin relative to the total number of residents within that specialty. The last row presents the total number of participants for each classification of origin relative to the total study population of whom a classification of origin was known (*n* = 491). Proportions in **bold** with an asterisk (*) indicate a significant adjusted residual in the analyses on whether the origin of respondents differed across the four groups of specialties. The country of birth of each specialism has been included as a footnote

	Dutch origin *n* (% Dutch origin / % of specialty)	Migrant *n* (% migrants / % of specialty)	Child of migrant(s) *n* (% children of migrant(s) / % of specialty)	Subtotal of Child of migrant(s) *n* (% children of migrant(s) / % of specialty)			
				1 parent inside Europe	1 parent outside Europe	2 parents inside Europe	2 parents outside Europe
Surgical^a^ (*n* = 120)	71* (21.2 / 59.2)	25* (37.9 / 20.8)	24 (26.7 / 20.0)	2 (11.1 / 1.7)	8 (27.6 / 6.7)	0 (0.0 / 0.0)	14 (35.0 / 11.7)
Internal medicine^b^ (*n* = 159)	112 (33.4 / 70.4)	15 (22.7 / 9.4)	32 (35.6 / 20.1)	7 (38.9 / 4.4)	12 (41.4 / 7.5)	1 (33.3 / 0.006)	12 (30.0 / 7.5)
Diagnostic/ supportive^c^ (*n* = 47)	38* (11.3 / 80.9)	5 (7.6 / 10.6)	4 (4.4 / 8.5)	1 (5.6 / 2.1)	1 (3.4 / 2.1)	1 (33.3 / 2.1)	1 (2.5 / 2.1)
Family & intellectual disability medicine^1, d^ (*n* = 167)	114 (34.0 / 69.1)	21 (31.8 / 12.7)	30 (33.3 / 18.2)	8 (44.4 / 4.8)	8 (27.6 / 4.8)	1 (33.3 / 0.6)	13 (32.5 / 7.9)
TOTAL (*n* = 493)	335 (68.2)	66 (13.4)	90 (18.3)	18 (3.7)	29 (5.9)	3 (0.006)	40 (8.1)

^1^ Data on the classification of origin from two respondents of the specialty family medicine & intellectual disability medicine are missing due to an incorrectly or incompletely provided answer to the question

Note: Definitions are based on the new population classification by origin of the Statistics Netherlands [21]. *Residents* with a Dutch origin are individuals who were born in the Netherlands and whose parents were also born in the Netherlands. A *migrant* is a person who was born outside the Netherlands. A resident who is a *child of migrant(s)* is an individual who was born in the Netherlands but has one or both parents born outside the Netherlands (with a distinction made inside or outside Europe)

*Country of birth for each specialism*

^a^ From the 120 surgical residents, 95 (79.2%) were born in the Netherlands, 10 were born in Europe (8.3%), and 15 (12.5%) were born outside Europe

^b^ From the 159 internal medicine residents, 144 (90.6%) were born in the Netherlands, 3 were born in Europe (1.9%), and 12 (7.5%) were born outside Europe

^c^ From the 47 diagnostic/supportive residents, 42 (89.4%) were born in the Netherlands, two were born in Europe (4.3%), and 3 (6.4%) were born outside Europe

^d^ From the 167 residents from family medicine & intellectual disability medicine,145 (86.8%) were born in the Netherlands, 6 were born in Europe (3.6%), and 15 (9.0%) were born outside Europe

**Table 3:** An overview of the distribution of the classification by origin of respondents, stratified by ethnic background. The last row represents the percentages based on the total number of respondents with a known classification of origin who provided an answer to these questions (*n* = 489 for the question on completion of higher education and *n* = 490 for the question on working as a medical doctor)

	Parent(s)/guardian(s) completed higher education^1^ (*n*, % of ethnic background)		Parent(s)/guardian(s) working as a medical doctor^2^ (*n*, % of ethnic background)	
Dutch origin	255	(76.3%)	42	(12.6%)
Migrant	52	(78.8%)	12	(18.2%)
Child of migrant	55	(61.8%)	14	(15.6%)
TOTAL	362	(74.0%)	68	(13.9%)

^1^ Answers from two respondents are missing, one of whom indicated that they did not know the answer to this question; ^2^ Answer from one respondent is missing

**Table 4A:** Median scores with the IQR (Q1-Q3), ranging from 1–10 with 10 being the highest, indicating how well the residents can identify with their supervisor^1,2^

	Dutch origin		Migrant		Child of migrant	
Surgical	8	[7–8]	7	[6–8]	7	[6-7.8]
Internal medicine	8	[7–9]	6	[5–8]	8	[6–8]
Diagnostic/supportive	8	[8–9]	6	[6–9]	9	[7.3–10]
Family & intellectual disability medicine	8	[7–8]	5	[3.5-8]	7	[5–8]
TOTAL	8	[7–9]	7	[5–8]	7	[5–8]

^1^ Data from three respondents in total are missing due to an incorrectly provided or missing answer to the questions

^2^ In total, 10 (8.3%) residents from surgical specialties, 19 (11.9%) residents from internal medicine specialties, 8 (17.0%) residents from diagnostic/supportive specialties, and 18 (10.8%) residents from family medicine & intellectual disability specialties provided a score of 10/10. Therefore, they did not receive the second question on the level of resemblance

**Table 4B:** The number of residents who primarily identify as being of Dutch origin, accompanied by the percentage within each specialty and their corresponding classification of origin^1^. The last row represents the total number of respondents who primarily identify as being of Dutch origin relative to the total number of respondents in each classification of origin (*n* = 335, *n* = 66, and *n* = 90 for Dutch origin, migrant, and child of migrant, respectively)

	Dutch origin		Migrant		Child of migrant	
Surgical	67	(94.4%)	14	(56%)	18	(75%)
Internal medicine	111	(99.1%)	6	(40%)	25	(78.1%)
Diagnostic/supportive	38	(100%)	0	(0%)	3	(75%)
Family & intellectual disability medicine	112	(98.2%)	5	(23.8%)	22	(73.3%)
TOTAL	328	(97.9%)	25	(37.9%)	68	(75.6%)

^1^ Data from three respondents in total are missing due to an incorrectly provided or missing answer to the question

### Total study population

In the total study population, residents experienced significant differences with their supervisors on an ethnic level (χ^2^ (df = 2, *N* = 491) = 59.2, *P* < 0.001, *V* = 0.35 (moderate effect)). This observation is mainly driven by a lower proportion of residents of Dutch origin and a higher proportion of migrants and children of migrant(s) reporting experiencing ethnic differences than expected. In total, 6.0% of residents of Dutch origin experienced such differences on an ethnic level (ASR − 7.7) *versus* 31.8% of residents who are migrants (ASR 4.4) and 32.2% of residents who are children of migrant(s) (ASR 5.4).

Also, significant differences were felt on a cultural level (χ^2^ (df = 2, *N* = 491) = 70.7, *P* < 0.001, *V* = 0.38 (moderate effect)) in the total study population. This was mainly driven by a lower proportion of residents of Dutch origin (24.2%, ASR − 8.0) and a higher proportion of migrants (72.7%, ASR 6.7) and children of migrant(s) (53.3%, ASR 3.8) reporting such differences than expected.

**Fig. 1:**
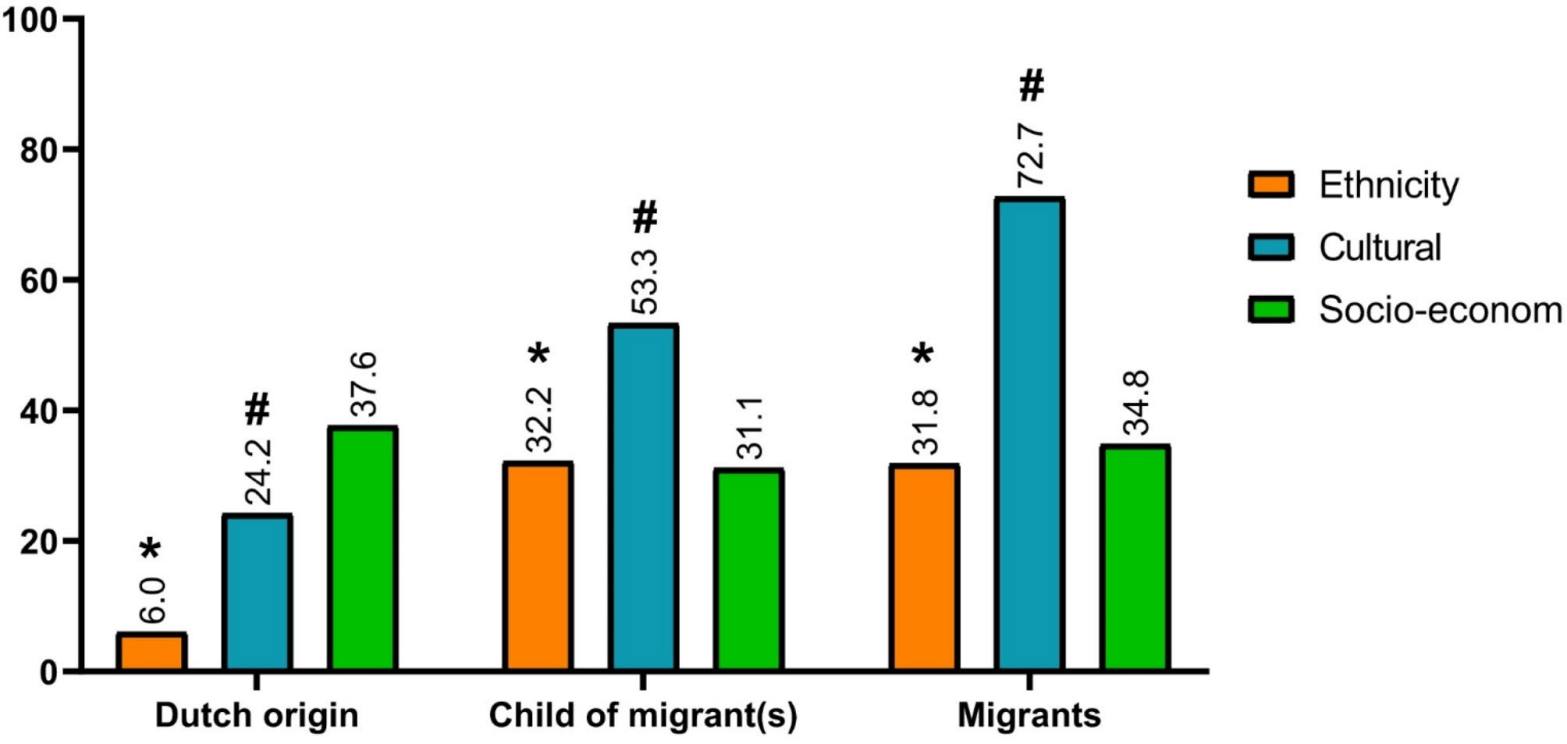
Bar plot illustrating the percentage of residents reporting differences with their supervisor(s) on an ethnic, cultural, and socio-economic level in the total study population. These plots are stratified by the residents’ classification of origin. To identify differences across classifications of origin, statistical analyses were conducted with stratification by levels on which discrepancies were experienced (i.e. ethnic, cultural, and/or socio-economic). An asterisk (*) above a bar indicates a significant adjusted residual for ethnicity and a number sign (#) above a bar indicates a significant adjusted residual for cultural differences

Yet, overall, residents experienced no significant differences with their supervisors on a socio-economic level (χ^2^ (df = 2, *N* = 491) = 1.3, *P* = 0.513, *V* = 0.05) (all percentages are also presented in Fig. 1).

### Surgical specialties

**Fig. 2:**
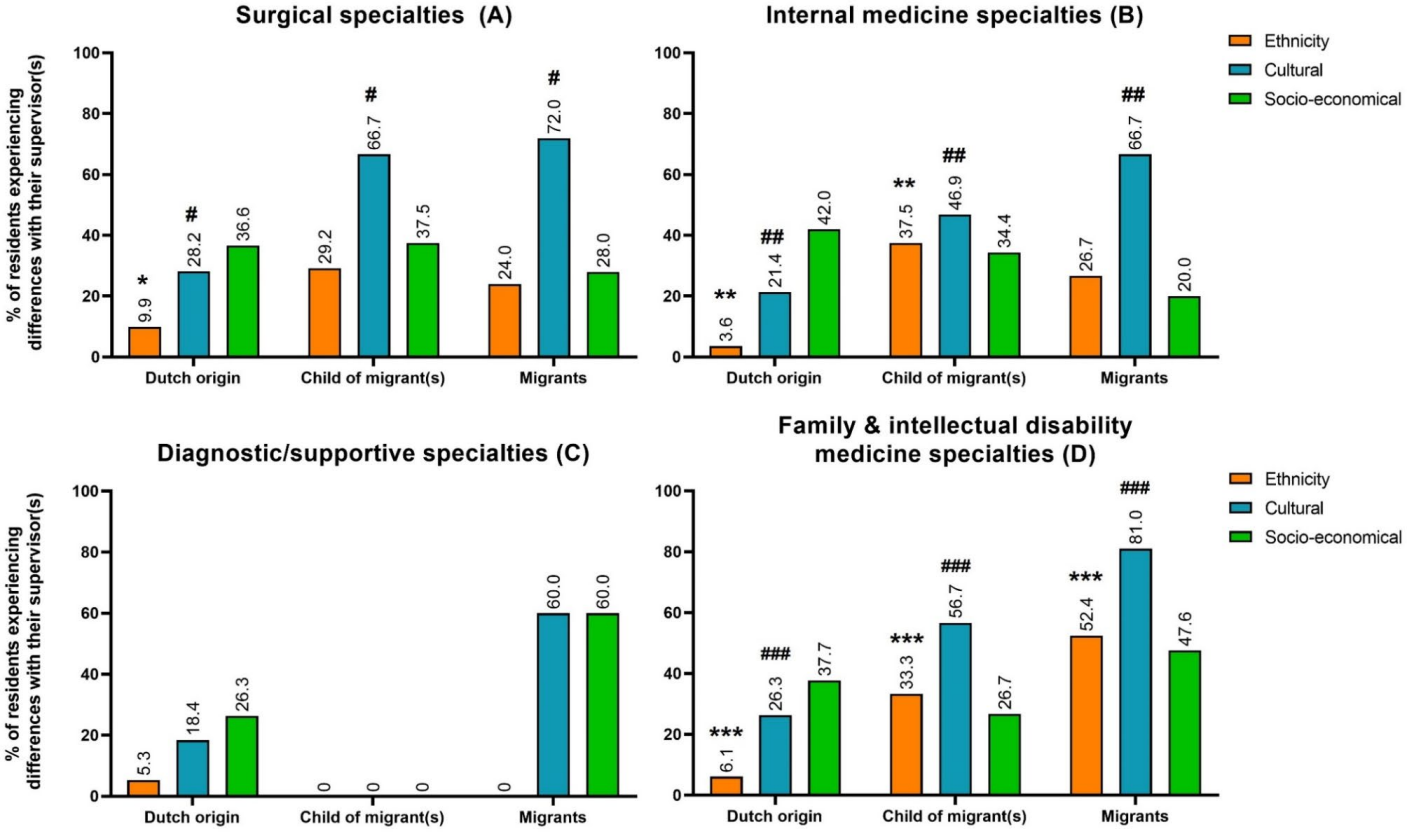
Bar plots illustrating the percentage of residents reporting differences with their supervisor(s) on an ethnic, cultural, and socio-economic level. These plots are stratified by specialty (**A-D**) and the residents’ classification of origin. To identify differences across classifications of origin, statistical analyses were conducted with stratification by (i) specialty and (ii) levels on which discrepancies were experienced (i.e. ethnic, cultural, and/or socio-economic). An asterisk (*) above a bar indicates a significant adjusted residual for ethnicity and a number sign (#) above a bar indicates a significant adjusted residual for cultural differences. The number of asterisks and symbols represents the different groups being compared; therefore, it is important to note that the quantity of symbols does not correspond to the significance level of the adjusted residual

Surgical residents experienced significant differences with their supervisors on an ethnic level (χ^2^ (df = 2, *N* = 120) = 6.0, *P* = 0.048, *V* = 0.22 (moderate effect)), mainly driven by a lower proportion of residents of Dutch origin that experienced differences than expected. In total, 9.9% of these surgical residents of Dutch origin experienced such differences (ASR − 2.4). Also, differences were felt on a cultural level (χ^2^ (df = 2, *N* = 120) = 20.0, *P* < 0.001, *V* = 0.41 (relatively strong effect)), mainly driven by a larger proportion of migrants (72%, ASR 3.0) and children of migrant(s) (66.7%, ASR 2.4), and a significantly smaller proportion of residents of Dutch origin (28.2%, ASR − 4.5) reporting such differences than expected. Yet, surgical residents experienced no significant differences with their supervisors on a socio-economic level (χ^2^ (df = 2, *N* = 120) = 0.7, *P* = 0.723, *V* = 0.08) (percentages also presented in Fig. 2A).

### Internal medicine specialties

Differences with their supervisor(s) were felt by these residents on an ethnic level (χ^2^ (df = 2, *N* = 159) = 29.0, *P* < 0.001, *V* = 0.43 (relatively strong effect)), mainly by a significantly higher proportion of children of migrant(s) (37.5%, ASR 4.8) and lower proportion of residents of Dutch origin (3.6%, ASR − 5.3) than expected, but not by migrants (26.7%, ASR 1.7). Significant differences were also present on a cultural level (χ^2^ (df = 2, *N* = 159) = 17.5, *P* < 0.001, *V* = 0.33 (moderate effect)), mainly reported by migrants (66.7%, ASR 3.2) and followed by children of migrant(s) (46.9%, ASR 2.2), while a significantly lower proportion of residents of Dutch origin did not experience such differences (21.4%, ASR − 4.0). In addition, no significant differences were felt by residents of internal medicine specialties on a socio-economic level (χ^2^ (df = 2, *N* = 159) = 3.0, *P* = 0.243, *V* = 0.14) (percentages also presented in Fig. 2B).

### Diagnostic/supportive specialties

No significant differences were felt by residents from supportive specialties with their supervisor(s) on an ethnic level (χ^2^ (df = 2, *N* = 47) = 0.5, *P* = 1.000, *V* = 0.10), cultural level (χ^2^ (df = 2, *N* = 47) = 5.7, *P* = 0.061, *V* = 0.35), or socio-economic level (χ^2^ (df = 2, *N* = 47) = 4.2, *P* = 0.168, *V* = 0.30) (percentages presented in Fig. 2C).

### Family medicine and intellectual disability medicine specialties

Significant differences with their supervisor were reported on an ethnic level (χ^2^ (df = 2, *N* = 165) = 33.9, *P* < 0.001, *V* = 0.45 (relatively strong effect)), mainly by a higher proportion of migrants (52.4%, ASR 4.6), followed by a higher proportion of children of migrant(s) (33.3%, ASR 2.6), but by a significantly lower proportion of residents of Dutch origin (6.1%, ASR − 5.5) than expected. Similarly, significant differences were felt on cultural level (χ^2^ (df = 2, *N* = 165) = 27.2, *P* < 0.001, *V* = 0.41 (relatively strong effect)), mainly by migrants (81.0%, ASR 4.2), followed by children of migrant(s) (56.7%, ASR 2.2), but by a significantly lower proportion of residents of Dutch origin (26.3%, ASR − 4.9) than expected. No significant differences between groups were experienced on socio-economic level (χ^2^ (df = 2, *N* = 165) = 2.4, *P* = 0.311, *V* = 0.12) (percentages also presented in Fig. 2D).

### Additional comments

While formal analysis of individual comments is beyond the scope of this paper, it is noteworthy that several residents (also) noted differences with their supervisors in the areas of: (i) age, life phase, life experience, and generational differences, (ii) gender, sexuality, and sexual preference, (iii) religion, worldview, and politics, and (iv) work-life balance, ambitions, and work ethics.

Additional noteworthy answers and comments provided by the residents are summarized in the Supplemental Materials file 1, showing a great variation in experiences, perceptions, and opinions regarding diversity in medical training and the workplace, with some noting significant ethnic diversity. In contrast, others highlight barriers due to the lack of diversity and underrepresentation of certain groups, including those with a migration background or different cultural practices. Also, several residents mentioned that putting further emphasis on diversity is unnecessary, arguing that candidates for specialization training should be chosen based on their excelling qualities and characteristics, regardless of any gender, race, or origin.

## Discussion

This survey study in the largest academic hospital in the Netherlands showed that the percentage of residents who are (children of) migrants is not an adequate representation of its highly multicultural surrounding area. Yet, this cohort of residents adequately reflects the demographics of the Netherlands as a whole. Interestingly, notable differences between the four groups of medical specialties existed. Surgical specialties showed the highest degree of diversity, while internal medicine and diagnostic/supportive specialties showed the lowest level of ethnic diversity.

Additionally, we observed that in general, the less represented groups – in ascending order from residents of Dutch origin, to children of migrant(s), and then migrants – reported more frequently that they experienced differences with their supervisor(s) on an ethnic or cultural level, however not on a socio-economic level.

The limited diversity across medical specialties and their residents is an observation supported by previous literature [15, 22, 23]. With the percentage of residents of Dutch origin in our study ranging from 59.2 to 80.9%, none of the specialties reflected the ethnic diversity present in the area surrounding the Erasmus MC. Overall in the Netherlands, the percentage of inhabitants of Dutch origin was 74.8% in 2022 [2], which falls within the range we found in our cohort. However, in the highly multicultural city of Rotterdam and surrounding area, where the majority of the medical students at Erasmus MC come from, the numbers are very different. In 2024, 43.5% of the inhabitants of Rotterdam are of Dutch origin (44.5% in 2023), while 12.9% have an European background (12.6% in 2023) and 43.4% had a non-European background (42.9% in 2023) [20].

It is noteworthy that our data indicate no substantial loss in ethnic diversity in the transition from medical student to the residency state in the entire cohort, considering that a previous cross-sectional study conducted at the Erasmus MC revealed that 34.4% of first-year and 28.6% of final-year medical students were children of migrant(s) or migrant(s) [24]. Therefore, in that study, 65.6% of first-year and 71.4% of final-year medical students were of Dutch origin, which is in accordance with our results including an overall percentage of 68.2% of residents of Dutch origin. In this perspective, it is also important to realize that not all residents have completed their medical study at the Erasmus MC/University, as applications into residency programs are accepted from residents that have studied at other universities within the Netherlands as well. Also, to the best of our knowledge, medical students of most of other universities (except for the VU University Medical Center Amsterdam) are less ethnically diverse than medical students at the Erasmus MC.

We also compared our results with data from a retrospective cohort study of Mulder et al. on the later stages of becoming a registered medical specialist [15]. In that study, an anonymized, non-public register of healthcare professionals (including medical specialists) was used (known as the ‘BIG register’), to identify which demographic characteristics of first-year medical students are linked to their eventual registration as physicians [15].Their analyses of specialists with an active BIG registration in 2021 revealed that: (i) 55.4–81.7% of surgical specialists, (ii) 68.8–86.7% of internal medicine specialists, (iii) 66.0-86.2% of diagnostic/supportive specialists, and (iv) 83.5–88.2% of family medicine & intellectual disability medicine specialists had no migration background, and, thus, were of Dutch origin. In our study, the proportion of residents of Dutch origin in surgery (59.2%) and in internal medicine (70.4%) seems slightly lower than in the previous-mentioned cohort, while in diagnostic/supportive specialties the percentage of residents with Dutch origin (80.9%) is within the same range as in the cohort of medical specialists from that study. Nevertheless, particularly the substantially lower percentage of residents of family medicine and intellectual disability medicine specialties of Dutch origin (69.1%) in our study compared to the previously mentioned range based on national data is remarkable. While we cannot rule out some kind of response bias, this might indicate more barriers for residents who are (children of) migrants while in training to become a registered general practitioner or intellectual disability medicine specialist. This hypothesis is supported by a Dutch retrospective cohort study, which included trainees from general practitioner specialty training institutes [25]. The study showed that those from an ethnic minority, migrants in particular, were significantly more likely to face underperformance assessments than the majority group. In addition, considering the limited representativeness of students with a migration background eligible to study medicine compared to society as a whole, this loss of diversity may also occur at an earlier stage (i.e., during the transition to high school) [14].

It is, however, important to underline that our data reflect only the distribution of residents of the Erasmus MC in 2023, and are based on the newest classifications of ethnic background that have changed over the past years. Also, we included a broader range of specialties than Mulder et al. Moreover, due to our cross-sectional study design, we cannot draw conclusions about the percentage of residents who ultimately obtain registration in the medical specialties for which they are currently in training. Nevertheless, our data from the current cohort of residents at Erasmus MC in 2023 may indicate a somewhat more ethnically diverse population compared to the previously described cohort of medical specialists, which included both young professionals, but also those nearing retirement. This shift likely reflects the growing and positive impact of increased awareness and initiatives aimed at promoting diversity.

Although we did not present distributions stratified for each individual specialism to avoid traceability to individuals, our findings on differences between specialties are at least partly supported by international data. We observed the highest degree of diversity in surgical specialties, followed by the specialties family & intellectual disability medicine. A previous study from the United States also showed that family medicine had the greatest representation of applicants from racial and ethnic groups underrepresented in medicine [26]. Deville et al. assessed the graduate medical education diversity in the United States, showing a significantly decreased representation of blacks and Hispanics in radiology, orthopedic surgery, and otolaryngology [27]. However, a recent cross-sectional study showed that postgraduate trainees in the United States from ethnic groups underrepresented in medicine (such as African American, American Indian, Hispanic, etc., excluding Asian and White groups and those classified as other/unknown) were less commonly represented in surgical specialties than in non-surgical specialties [23]. The higher degree of diversity in surgical specialties observed in our study might be explained by a combination of factors. First, male residents from certain ethnic minorities, including Middle Eastern and Asian men, may have a preference for pursuing careers in surgery, for example due to the perceived prestige or financial reasons [28]. Second, we observed the lowest proportion of female residents in surgical specialties. As previously reported, gender bias could contribute to the “hidden curriculum” (i.e., unwritten rules or perspectives of the learning process) and vice versa, placing women at a disadvantaged position [29]. This gender bias might further amplify the contribution of the first factor, attracting more men from ethnic minorities while simultaneously creating an environment that is less appealing to women (from ethnic minorities).

Our results on the perceived level of resemblance or “items of inclusivity” underline that better scores on these items are only partially linked to increased diversity among medical residents. In general, substantial differences between residents and their supervisor(s) were experienced on ethnic and cultural levels. While surgical specialties had the highest level of diversity, substantial differences were still felt by children of migrant(s) and migrants. It is also remarkable that, across all specialties, a significant number of residents of Dutch origin still reported experiencing differences with their supervisors on cultural and socio-economic levels. This highlights the potential importance and role of diversity factors beyond ethnicity, including but not limited to sex and gender – especially considering the high female-to-male ratio of residents in our cohort and the recently demonstrated advantageous position of females without a migration background in becoming physicians [15]. In addition, considering that we specifically asked about perceived differences with supervisors, the observed increased diversity among medical residents does not necessarily reflect the level of diversity among medical staff – which is an important determinant for sense of belonging and inclusivity.

Furthermore, despite the small differences in the proportion of having a higher-educated parent(s)/guardian(s) across ethnicities, and the absence of differences in the proportion of parent(s)/guardian(s) working as medical doctors, migrants had the highest percentage of parent(s) or guardian(s) with higher education or working as medical doctors. Although causal conclusions cannot be drawn from this observation, it could be hypothesized that our study population contains a selected group of migrants with higher educated parent(s) or guardian(s), considering the already relatively lower likelihood of migrants becoming a medical specialist [15]. Indeed, previous studies have consistently demonstrated that having a favorable socio-economic background, including high-educated parents with a high income as well as parent(s) registered as healthcare professionals, positively influences admission, performance and eventual odds of becoming a medical specialist [15, 30, 31]. Such cumulative advantages are also named the “Matthew effect” [32].

### Limitations and strengths

An important limitation of our study is the potential for selection bias, with 50% of the residents responding – a group that might be more motivated to participate in a study about diversity and inclusion. As we had no information on the distribution of non-responders across different medical specialties and origins, we cannot determine the extent of this potential selection bias. Second, these respondents had already passed the stringent selection procedures required for specialization training. Thus, our data do not account for the effort, pre-existing advantageous circumstances or the extent of the “Matthew effect” that facilitated their acceptance to these positions. Third, children of migrants and migrants included in our study might not represent those not accepted into these specialization trainings. Indeed, a previous qualitative study showed that doctors from immigrant backgrounds often consciously distance themselves from minority groups, having been taught since childhood the importance of assimilating into the majority culture [33]. Fourth, as our question on perceived level of resemblance did not contain any further explanation, the differentiation between the terms “cultural” and “ethnicity” could have been confusing for some respondents. Fifth, while the response was distributed equally among the surgical, internal medicine, and family medicine & intellectual disability medicine specialties, residents from diagnostic/supportive specialties were less represented without evident reasons. In addition, to ensure the anonymity of respondents, we could not present the representation of individual ethnicities within specific specialties. Lastly, a non-validated questionnaire was used to answer the research questions.

Nevertheless, as far as we know, we studied the current degree of ethnic diversity of a Dutch cohort of medical residents for the first time. Although our findings highlight the underrepresentation of non-Dutch residents, particularly in light of our highly multicultural region, we did not observe a substantial loss of ethnic diversity in the transition from medical student to residency. This suggests that the “leaky pipeline” phenomenon may occur at different stages of the medical career, which is an important new insight. Furthermore, our additional questions on “inclusivity” enrich the existing literature, which often lacks such qualitative insights.

### Future research

We encourage the replication of this study in other academic centers and countries and emphasize the need for these studies to address aforementioned limitations, including the potential for selection bias and the use of a non-validated questionnaire. Indeed, it is essential to enhance diversity and inclusiveness in the workforce and learning environment in healthcare, including hospitals, for several reasons related to enhancing patient care and promoting equal opportunities for medical students, regardless of their ethnic background. Considering earlier concerns about the limited transition of ethnically diverse medical students into residency programs, future research should focus on different factors influencing this career step. These factors include the recruitment and selection procedures as well as inclusiveness and social safety during residency programs, which contribute to improved academic performance, influencing sense of belonging and mental and somatic well-being [34]. Additionally, previous studies indicate that students from racial and ethnic minority groups are more likely to apply for specialties with greater representation of their racial or ethnic group among practicing physicians [26]. Therefore, future studies and interventions to enhance the representation and diversity among medical specialists are needed. Importantly, awareness and recognition of similarity bias, implicit bias, stereotyped thinking patterns, and (unconscious) cultural or exclusionary workplace practices are crucial [15]. A subsequent step involves (i) implementing transparent, diverse, and inclusive recruitment and selection policies, and (ii) evaluating whether such interventions lead to improved well-being, less study delay, lower drop-out rates, and ultimately, better progression towards becoming a medical specialist. Lastly, while we mainly focused on ethnic identities, future studies in which intersectionality is implemented are pivotal. This involves examining how ethnic identities interact or intersect with other identities, including sex identity, gender identity, cultural identity, religion, age, and disability or chronic illness [35].

## Conclusions

In our cohort of current Dutch residents we showed no substantial loss of ethnic diversity in the transition from medical student to residency, although notable differences were observed across medical specialties with the highest degree of diversity in surgical specialties. However, the percentage of residents who are (children of) migrants is not an adequate representation of the multicultural region of Rotterdam, suggesting the “leaky pipeline” phenomenon occurs mostly at earlier career stages. Overall, residents who are (children of) migrants score lower on inclusivity items. Importantly, there is no clear association between level of diversity and perceived level of inclusion. To improve sense of belonging during residency trainings and fairness and equity in the transition of young medical doctors to becoming a medical specialist, further intersectional research is warranted.

## Electronic supplementary material

Below is the link to the electronic supplementary material.

Supplementary Material 1

Supplementary Material 2

## Data Availability

Data is provided within the manuscript or supplementary information files.
