# Supplementary material for: Ethnic diversity and inclusiveness among medical residents in the Netherlands: results from a single-centered survey study
Source: BMC Med Educ. 2025 Feb 25;25:308. doi: 10.1186/s12909-025-06878-5 (PMC11863459; doi:10.1186/s12909-025-06878-5)
Supplement: Supplementary file 1 — Supplementary Material 1 [file 12909_2025_6878_MOESM1_ESM.docx]

**SUPPLEMENTAL MATERIALS FILE 1**

Residents were asked the open question: “What else do you want to say or add about diversity in the medical residency program?”. The responses selected below offer an impression of the typical and average responses, irrespective of the origin or specialty of the resident.

- “I am of the opinion that people with the best qualities/characteristics – regardless of gender, race, or origin – should be selected for any training whatsoever. In other words, it is a bad thing to favor people based on gender, race, or origin. Therefore, I am of the opinion that the 'diversity doctrine' should stop.” – *Male resident from an internal medicine specialty*
- “In my experience, diversity is not just about gender, age, ethnicity, and educational level. But also about religion, sexual diversity, culture, and physical capabilities. I am currently missing that in this questionnaire.” – *Female resident from the specialties family medicine or intellectual disability medicine*
- “From my own experience, I know that people look differently (especially) at a woman of another color or wearing a headscarf. They more quickly assume that you might be more like a housewife/stay-at-home, have many children, etc.” – *Female resident from an internal medicine specialty*
- “In my view, the training is enormously ethnically diverse.” – *Male resident from a supportive specialty*
- “During the clerkships, I often saw and experienced that students with a migration background are assessed differently/worse than students without a migration background. Among ourselves, we often mentioned this and exchanged stories, and it was almost seen as 'normal'. It is very difficult to discuss this with students without a migration background, or with the concerning medical specialist without a migration background. The difference is not seen.” – *Female resident from the specialties family medicine or intellectual disability medicine*
- “[There is] too little diversity due to insufficient good progression from medical education to medical postgraduate training. This is due to opaque application and selection procedures, and thereby, I think, discouraging many people who think they do not fit in.” – *Female resident from a surgical specialty*
- “In [a surgical specialty], diversity is good as far as I am concerned. About 50% of the residents have at least one parent who is not from the Netherlands. Furthermore, this is also well represented in the staff. What is notable, however, is that the number of women is still relatively low compared to other specialties.” – *Male resident from a surgical specialty*
- “It is very segmented. I have worked in other countries including UK, and the Netherlands is by far one of the most exclusive country regarding diversity especially in the medical field. Look at any hospital department website and the photos of specialist says it all whilst there are many doctors with diverse culture who are eliminated from work opportunities due to this.” – *Female resident from the specialties family medicine or intellectual disability medicine*
- “In [the specialties family medicine or intellectual disability medicine], residents come from many diverse backgrounds, in sharp contrast to what you see in other popular medical specialties (e.g., Surgery, Gynecology, etc.)” – *Male resident from the specialties family medicine or intellectual disability medicine*
- “There is clearly little diversity, and the people who are accepted for the medical postgraduate training (of which I am one) are, in my opinion, people who require little 'adjustment' from the majority. So, for example, people with a migration background wearing a headscarf, who do not drink alcohol, etc., have more difficulty getting into training than people with a migration background who, for example, can go to that one party [afternoon drinks] and drink alcohol.” – *Female resident from an internal medicine specialty*
- “Diversity is low. Admission requirements for the program are that you must speak Dutch and have followed the correct pre-education. An education followed abroad is not automatically approved, and I think that is a hurdle to hiring someone with a foreign diploma.” – *Female resident from a supportive specialty*
