# Supplementary material for: Ethnic diversity and inclusiveness among medical residents in the Netherlands: results from a single-centered survey study
Source: BMC Med Educ. 2025 Feb 25;25:308. doi: 10.1186/s12909-025-06878-5 (PMC11863459; doi:10.1186/s12909-025-06878-5)
Supplement: Supplementary file 2 — Supplementary Material 2 [file 12909_2025_6878_MOESM2_ESM.docx]

**SUPPLEMENTAL MATERIALS FILE 2 – Study Questionnaire**

*Questions marked with an asterisk (*) are mandatory to complete the questionnaire.*

1. What is your age?* *(Only numbers allowed)*

Answer: ______ years

1. What specialty are you currently training for?* *(Select all options that apply)*

- Anesthesiology
- Cardiology
- Cardiothoracic Surgery
- Dermatology
- Intellectual disability medicine
- Gynecology & Obstetrics
- General Surgery
- General Practice (Family Medicine)
- General Internal Medicine
- Oral and Maxillofacial Surgery
- Otorhinolaryngology (ENT - Ear, Nose, and Throat)
- Pediatrics
- Clinical Chemistry
- Clinical Physics
- Clinical Genetics
- Clinical Geriatrics
- Pulmonary Diseases and Tuberculosis
- Gastroenterology
- Medical Microbiology
- Neurosurgery
- Neurology
- Nuclear Medicine
- Ophthalmology
- Orthopedics
- Pathology
- Plastic Surgery
- Psychiatry
- Radiology
- Radiotherapy
- Rheumatology
- Emergency Medicine
- Urology
- Hospital Pharmacy
- Other specialism, namely: ______

1. What year did you start your training to become a medical specialist?* *(Only numbers allowed)*

In the year: ______

1. What is your country of birth?*

Answer: ______

*Note: This specifically concerns the country,* ***not*** *the continent.*

1. What is your mother's country of birth?*

Answer: ______

*Note: This specifically concerns the country,* ***not*** *the continent. Please enter "unknown" if you do not know or if it is unknown.*

1. What is your father's country of birth?*

Answer: ______

*Note: This specifically concerns the country,* ***not*** *the continent. Please enter "unknown" if you do not know or if it is unknown.*

1. Which ethnicity or ethnicities do you identify with? You may enter multiple ethnicities. If you identify with more than one, please rank them starting with the ethnicity you identify with the most and ending with the one you identify with the least.* *(At least one answer is required)*

- Ethnicity 1: ______
- Ethnicity 2 (if applicable): ______
- Ethnicity 3 (if applicable): ______
- Ethnicity 4 (if applicable): ______
- Ethnicity 5 (if applicable): ______

*For example: 1. Dutch and 2. Turkish* ***or****: 1. Moroccan, 2. Dutch, 3. German*

1. To what extent do you identify with your supervisors at work? This question relates to your (subjective) experience.*


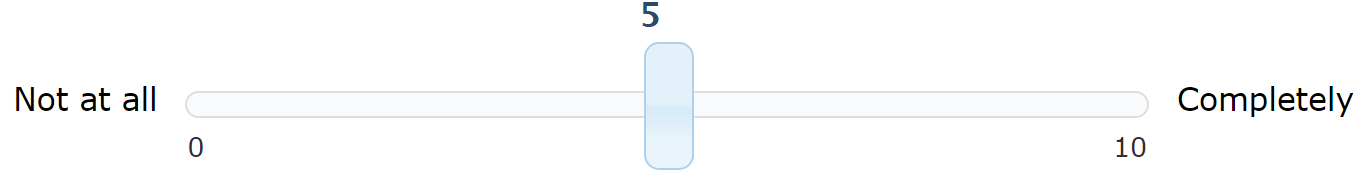


*Move the vertical slider to the point on the scale that, in your opinion, indicates the extent to which you identify with your supervisors at work.*

If a score of less than 10 was given for question 9, question 10 will be asked.

1. In which area (or which areas) do you experience differences?*

- Ethnicity
- Culture
- Socio-economic
- Other, namely: ______

*Multiple answers are possible.*

1. Did at least one of your parents/guardians complete higher education (higher professional education or university or equivalent foreign education)?* *(One answer allowed)*

- Yes
- No
- I do not know

1. Has at least one of your parents/guardians (ever) worked as a doctor?* *(One answer allowed)*

- Yes
- No
- I do not know

1. What is your gender?* *(One answer allowed)*

- Male
- Female
- X
- Prefer not to say

1. What would you like to share about diversity in medical training?

Answer: ______

**Many thanks for your participation in the study!**
